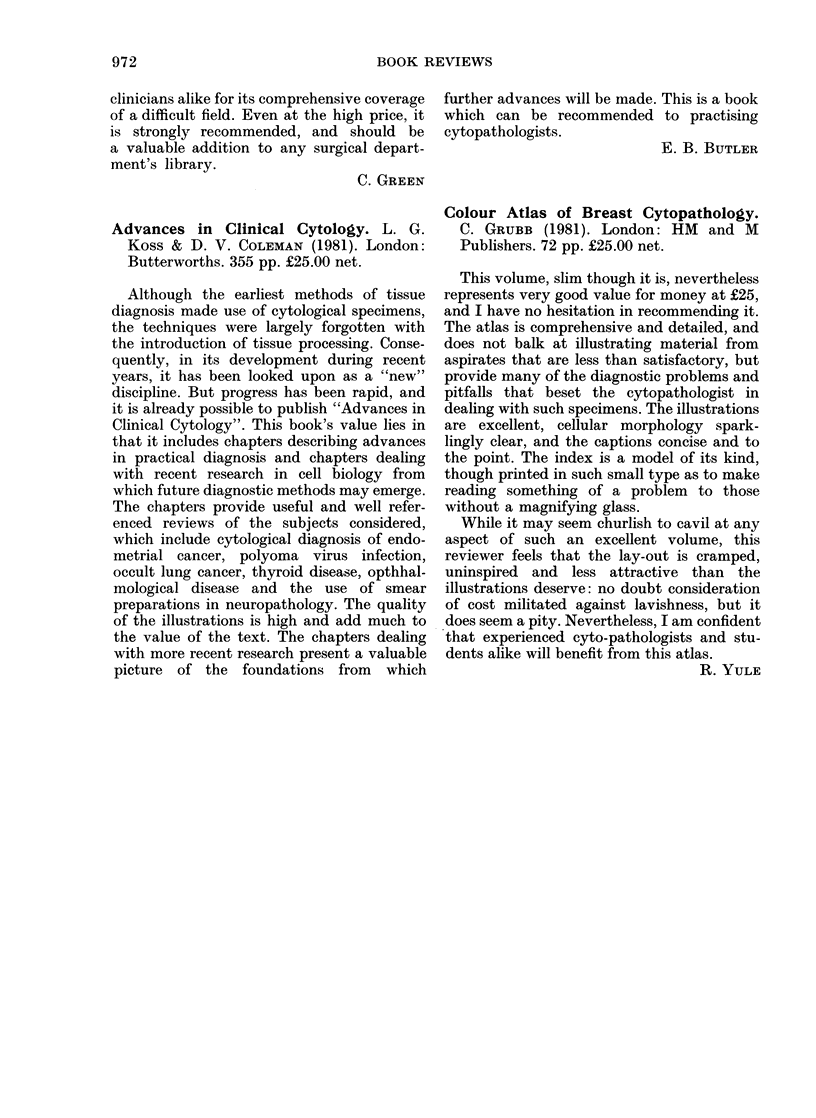# Colour Atlas of Breast Cytopathology

**Published:** 1982-06

**Authors:** R. Yule


**Colour Atlas of Breast Cytopathology.** C. GRUBB (1981). London: HM and M Publishers. 72 pp. £25.00 net.

This volume, slim though it is, nevertheless represents very good value for money at £25, and I have no hesitation in recommending it. The atlas is comprehensive and detailed, and does not balk at illustrating material from aspirates that are less than satisfactory, but provide many of the diagnostic problems and pitfalls that beset the cytopathologist in dealing with such specimens. The illustrations are excellent, cellular morphology sparklingly clear, and the captions concise and to the point. The index is a model of its kind, though printed in such small type as to make reading something of a problem to those without a magnifying glass.

While it may seem churlish to cavil at any aspect of such an excellent volume, this reviewer feels that the lay-out is cramped, uninspired and less attractive than the illustrations deserve: no doubt consideration of cost militated against lavishness, but it does seem a pity. Nevertheless, I am confident that experienced cyto-pathologists and students alike will benefit from this atlas.

R. YULE